# Controlled release from protein particles encapsulated by molecular layer deposition

**DOI:** 10.1039/c5cc03232f

**Published:** 2015-06-23

**Authors:** Siddarth A. Vasudevan, Yaolin Xu, Saurabh Karwal, Helena G. M. E. van Ostaay, Gabriel M. H. Meesters, Mojgan Talebi, Ernst J. R. Sudhölter, J. Ruud van Ommen

**Affiliations:** a Department of Chemical Engineering, Delft University of Technology The Netherlands J.R.vanOmmen@tudelft.nl

## Abstract

Molecular layer deposition (MLD) was used to coat micron-sized protein particles in a fluidized bed reactor. Our results show that the dissolution rate of particles coated *via* MLD rapidly decreases with the increase in number of coating cycles, while the uncoated particles dissolve instantaneously.

## Abstract 2

Molecular layer deposition was applied for coating micron-sized protein particles to control their dissolution behaviour.

## Graphical abstract

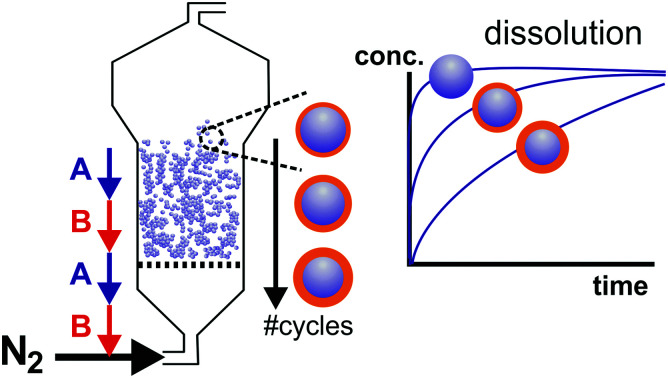

In the last two decades, much effort has been directed towards the development of drug delivery systems which overcome the shortcomings of the traditional methods such as toxic effects due to the unpredictable concentration levels, degradation of drugs in the digestive tract before entering the bloodstream, non-personalized nature, *etc.*^1–3^ With this development, proteins and peptides have become the natural choice for drugs due to their incredible specificity and bioactivity.^4,5^ However, their administration has been mostly limited to the parental route due to their low bioavailability.^6,7^ Encapsulation of the active pharmaceutical ingredient inside a shell is an attractive way to control the temporal and spatial release, by either varying the thickness or the composition of the coating.^8^ To this end, a number of novel and efficient drug delivery systems have been developed based on encapsulation methods.^9^ For certain applications, it would be attractive to have a full control over the coating thickness at the nm scale, while the coating is conformal.^10,11^ Such a method has a huge scope for extensibility to coating of bio-organic nanoparticles.^12^ Molecular layer deposition (MLD) can be used to achieve such a precise and well-controlled coating.

MLD is a thin-film growth technique developed during the early 1990s for the deposition of molecular fragments on the surface of an active material,^13^ and has been an attractive method for the deposition of a variety of organic polymers^14^ and more recently hybrid organic–inorganic polymers.^15^ In a typical MLD process, molecular fragments of the bi-functional precursors are deposited on the surface of an active substrate. This process involves two alternating reactions. It is the self-limiting nature of these reactions which enables the deposition of ultra thin layers on the surface of the substrate.^13^ In this communication, we show that controlled release of the active material can be achieved by MLD coating. To our knowledge, the work presented here is the first adaptation of the MLD process to encapsulate protein particles to study their controlled release properties.

To demonstrate the concept, we use protein particles as the substrate and the precursors used are malonyl chloride and 1,2-butanediol. An illustration of the reactions involved is shown in Fig. 1. The amine groups on the surface of the protein particles act as the active group for reaction with the acyl chloride group of malonyl chloride during the initiating reaction (reaction 0). In the next reaction step (labelled as reaction 1), the unreacted acyl chloride group of malonyl chloride, which is now attached to the surface of the substrate, acts as the active site for the reaction with the hydroxyl group of 1,2-butanediol. In 1,2-butanediol, the hydroxyl group at position 1 is the most reactive (less sterically hindered) in ester formation. In the subsequent reaction step (reaction 2), the unreacted hydroxyl group of 1,2-butanediol reacts with the acyl chloride group of malonyl chloride. Only the first coating cycle involves reaction steps 0 and 1, while subsequent coating cycles involve reaction steps 1 and 2.

**Fig. 1:**
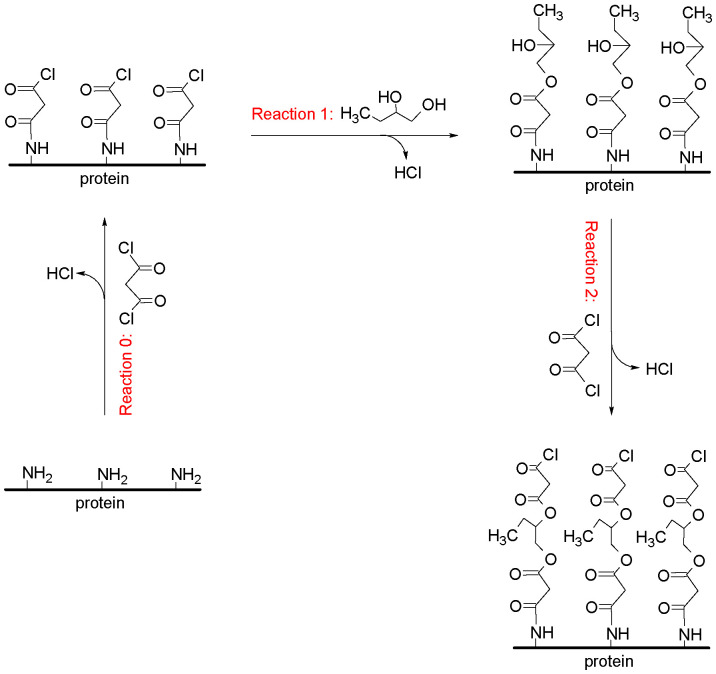
An illustration of the reactions involved in the MLD process.

2 g of protein particles with an average diameter of 200 μm are suspended in an upward gas flow of pure N_2_, which is called a fluidized bed. N_2_ acts as the carrier gas for feeding the precursors into the fluidized bed reactor (FBR). A schematic diagram of the experimental set-up is shown in Fig. 2.

**Fig. 2:**
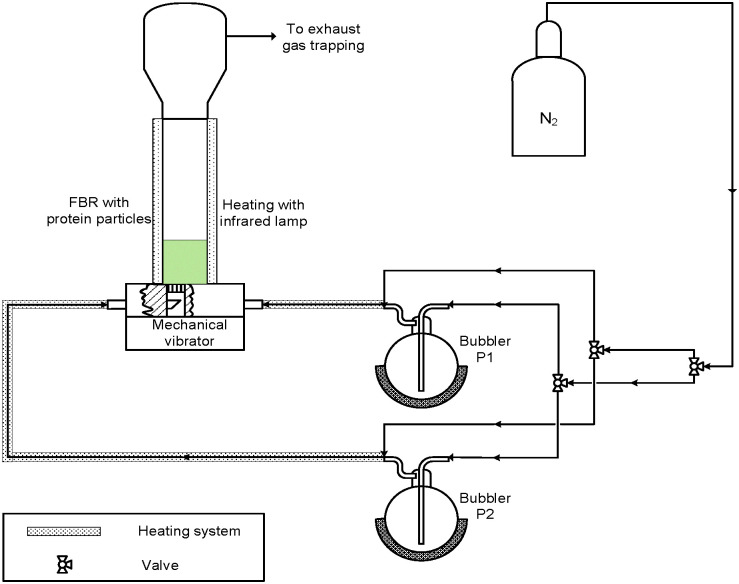
Schematic diagram of the experimental set-up used for the MLD process.

The FBR primarily consists of a vertical glass tube with an inner diameter of 2.6 cm and a length of 40 cm with thermocouples inserted at the entrance and exit. The FBR is maintained at a temperature between 40 °C and 45 °C using an infrared lamp. It is vital to maintain considerably low temperatures because the protein used is found to denature at a temperature above 55 °C. The denaturation temperature of the protein to a great extent limited the choice of the precursors for the MLD process. Because of the low vapour pressures of malonyl chloride and 1,2-butanediol at room temperature, they are preheated in bubblers to 40 °C and 115 °C, respectively. A distributor plate is provided at the entrance of the FBR to ensure a uniform distribution of the inlet gas stream mixture. By measuring the variation of the particle bed height with flow rate, the minimum velocity required to keep the particles afloat in N_2_ gas is determined to be 2.7 × 10^−2^ m s^−1^. This velocity is often referred to as the minimum fluidization velocity. The unreacted precursor and by-product of the reaction, HCl, is trapped using a mineral oil cold trap at the exit of the FBR. We employ two methods to improve the fluidization of the particles: the mechanical vibration of the FBR at a frequency of 50 Hz, and a microjet of 100 μm. The microjet is inserted into the FBR from the top and the mechanical vibrator is fixed at the bottom of the FBR. The microjet ensures good fluidization by breaking the agglomerates formed during the coating process.

Particles coated for different number of cycles are prepared. A typical coating cycle consists of four steps: 30 s dosage of malonyl chloride; minimum 2 min of purging with pure N_2_ to remove the unreacted precursor; 30 s dosage of 1,2-butanediol and finally purging with pure N_2_ for at least 2 min. The dosage times are relatively long (*i.e.*, much excess of the reactant is fed) to maximize completion of each cycle; this does not cause harm since the reaction is self-limiting. The purge times are chosen to be long (≫residence time) to prevent the unreacted compounds or products from staying behind in the tube system or on the particle surface. We observed a tendency for particle agglomeration during the reaction steps in a coating cycle, which is aggravated with increase in the number of cycles. This increased agglomeration affects the fluidization of the particles and in certain cases the fluidization is completely lost. Purging the FBR with high flow rate N_2_ gas for a long duration of time reinforced fluidization. Hence, for later cycles the reactor was purged until the fluidization was completely re-established. The particles were coated for 2, 6 and 10 MLD cycles. The particles showed an increasingly intense yellow/orange colour with the increasing number of cycles.

Fourier transform infrared (FTIR) spectroscopy has been used to characterize the coating of protein particles. The FTIR spectra are obtained using a Nicolet 8700 FTIR spectrometer (Thermo Electron Corporation) operated using a liquid N_2_ cooled KBr/DLaTGS D301 detector. The FTIR spectra of the coated protein particles are obtained by pressing the sample onto KBr salts and the data are collected with a resolution of 4 cm^−1^ averaged over 128 scans. The FTIR spectra of the coated particles shown in Fig. 3 are subtracted results from the spectra of the uncoated particles.

**Fig. 3:**
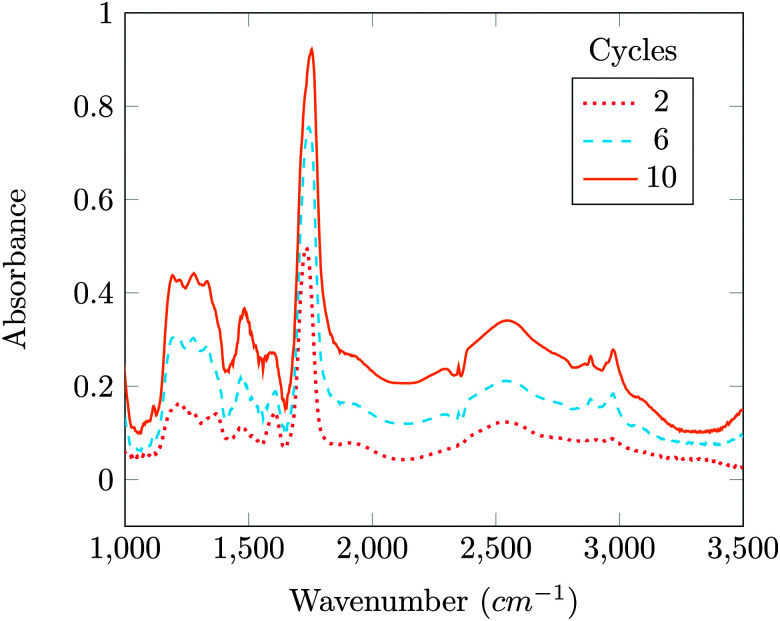
FTIR spectra for 2, 6 and 10 cycles samples. The data shown are difference spectra: the spectrum for the uncoated sample has been subtracted.

In Fig. 3, two distinct peaks are observed very close to 1736 cm^−1^ and 2971 cm^−1^; these correspond respectively to the stretching of [–COO–] and [CH_3_–] groups. Increase in the absorbance peak due to [–COO–] and [CH_3_–] stretching indicates the increase in the coating thickness with the number of cycles.

Dissolution experiments have been performed to study the controlled release of the coated protein samples. It is expected that during dissolution the ester bonds of the coating are stepwise hydrolysed. All the dissolution experiments have been performed at room temperature and pressure. 0.15 g of a coated particle sample is put in 150 ml of deionized water. The resulting mixture was stirred with a magnetic stirrer to ensure a uniform dispersion of the particles in deionized water. However, due to their low density most of the coated particles remain on the surface of the solution. Samples have been collected at regular intervals for a time period of 30 min. The collected samples are immediately filtered through a 0.45 μm pore size polyvinylidene difluoride membrane (Millex) to avoid further dissolution of protein particles. After a time period of 30 min, undissolved protein particles denatured to form strands in the solution.

UV-vis spectroscopic measurements (UV-1800, Shimadzu) were performed on the collected dissolution experiment samples of uncoated and coated protein particles at a wavelength of 260 nm. The results are shown in Fig. 4.

**Fig. 4:**
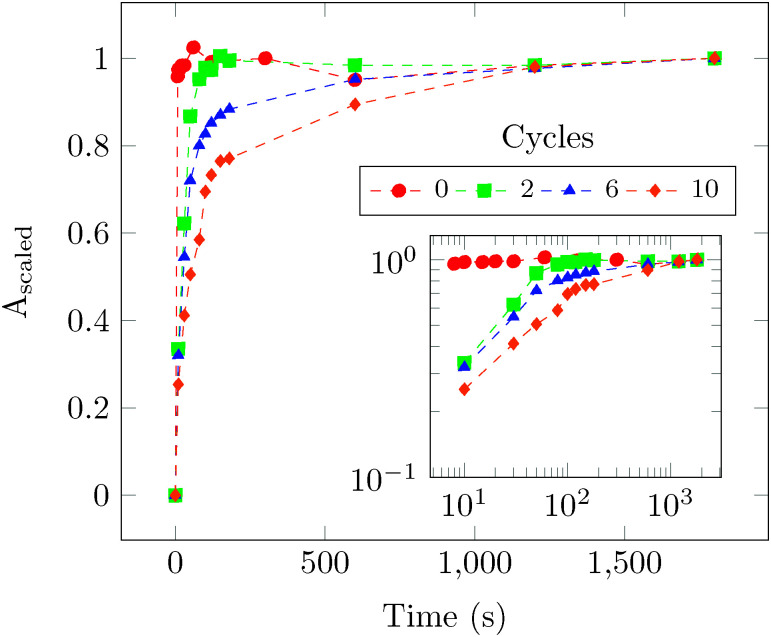
Dissolution results obtained using UV-vis spectroscopy for samples with different number of cycles.

In Fig. 4, *A*_scaled_ the scaled absorbance is defined as (*A*(*t*) − *A*(*t* = 0))/(*A*(*t* = 1800 s) − *A* (*t* = 0)), where *A*(*t*) is the absorbance at time *t* and *A*(*t* = 0) is assumed to be zero. Here, scaled absorbance gives a measure of scaled concentration because absorbance scales linearly with concentration. The solution containing uncoated samples attains a maximum concentration in about 10 s, while the coated samples dissolve at a much slower rate. In the inset of Fig. 4, the UV-vis test results are plotted on a log–log scale. Two distinct regions are observed: an initial short timescale of ∼30 s corresponding to a fast release of the coated protein and a long timescale ∼1000 s corresponding to a slow release of the coated protein.

Dissolution in controlled release applications is often described by a power law;^16^ we used this approach too. The fast and slow regions are fitted individually to a power law function which scales with time as *t*^*α*^. The values of *α*_fast_ for 2, 6 and 10 cycles samples, are respectively 0.482 ± 0.166, 0.425 ± 0.121 and 0.422 ± 0.059. *α*_fast_ values obtained are close to 0.5, which is observed in diffusion governed dissolution mechanism models.^16,17^ We suspect that the close resemblance of the fast release exponent to that of diffusion governed drug release mechanism models could be due to the presence of protein particles whose surface area is not completely coated. These particles are formed as a result of continuous breakage and formation of agglomerates, respectively, during purging and precursor dosage periods. For the 2 cycles sample, *α*_slow_ ≈ 0 indicating that the maximum concentration has been attained after a time period of 100 s. However, for 6 and 10 cycles samples, *α*_slow_ values are found to be 0.058 ± 0.008 and 0.117 ± 0.013, respectively. These exponents likely correspond to dissolution after de-esterification of the film.

In conclusion, we found that with an increasing number of coating cycles of MLD the thickness of coating increases as shown using FTIR. We also demonstrate experimentally that the controlled release of protein particles can be realized *via* MLD. The controlled-release behaviour is validated through the dissolution experiment of coated particles wherein the decrease in the rate of dissolution is observed with an increase in the number of coating cycles. This proof-of-principle demonstrates that MLD of fluidized particles is an attractive way to obtain protein materials with tunable controlled-release properties.

The research leading to these results has received funding from the European Research Council under the European Union’s Seventh Framework Programme (FP/2007-2013)/ERC Grant, agreement no. 279632, and the Royal Netherlands Academy of Arts and Sciences.
